# Association of Serotonin Transporter Gene Polymorphism with Recurrent Aphthous Stomatitis

**Published:** 2018

**Authors:** Shamsolmolouk Najafi, Mahsa Mohammadzadeh, Amirabbas Zahedi, Mansour Heidari, Nima Rezaei

**Affiliations:** 1. Faculty of Dentistry, Tehran University of Medical Sciences, Tehran, Iran; 2. International Campus, Dental Research Center, Tehran University of Medical Sciences, Tehran, Iran; 3. Dental Branch, Islamic Azad University, Tehran, Iran; 4. Farabi Eye Hospital, Farabi Research Center, Tehran University of Medical Sciences, Tehran, Iran; 5. Research Center for Immunodeficiencies, Children’s Medical Center, Tehran University of Medical Sciences, Tehran, Iran; 6. Department of Immunology, School of Medicine, Tehran University of Medical Sciences, Tehran, Iran; 7. Network of Immunity in Infection, Malignancy and Autoimmunity (NIIMA), Universal Scientific Education and Research Network (USERN), Boston, MA, USA

**Keywords:** *5-HTTLPR*, PCR, Recurrent aphthous stomatitis (RAS), Single nucleotide polymorphism

## Abstract

**Background:**

Recurrent Aphthous Stomatitis (RAS) is one of the most common diseases of the oral cavity all over the world (5–66%). RAS has a multifactorial etiology, while psychological factors such as stress and anger play a role in its manifestation. The serotonergic mechanisms particularly the serotonin-transporter gene (*5-HTT*) may affect the risk of psychological alterations and stress response. The aim of the present study was to evaluate the polymorphism of the promoter region of *5-HTT* (*5-HTTLPR*) in the patients with RAS, compared to that in the control subjects.

**Methods:**

In this case-control study, 100 patients with RAS and 100 healthy subjects were enrolled. PCR was performed on DNA of the samples, using a pair of primers capable of distinguishing S/L alleles and replicating *5-HTTLPR*.

**Results:**

No statistically significant difference existed between LL and LS genotype frequencies in the case and control groups. However, SS genotype frequency was significantly higher in the case group, as compared to the control group (p=0.001).

**Conclusion:**

The conclusion of the present study demonstrated that S allele could approximately double the risk of RAS.

## Introduction

One of the most common oral diseases all over the world is Recurrent Aphthous Stomatitis (RAS) ^1^. This disease is characterized by aphthous ulcers recurring in oral mucosa ^2^. Unlike erosion, loss of continuity involves the whole epithelium and may affect the underlying connective tissue ^3^. The prevalence of RAS varies greatly among different populations ^4,5^. According to recent studies, it is estimated to vary between 5 and 66% with a mean of 20% ^6^.

Although its exact etiology remains obscure, RAS is known to be a multifactorial disorder ^7^. Many local and systemic factors are related to RAS. Some studies have suggested that the defects of the immune system could associate with this disease ^8^. The investigations have indicated that RAS may have immunological, psychological, genetic, and microbial bases ^9,10^. For instance, the levels of depression, anxiety, and stress in the patients suffering from RAS were specified using Hamilton Anxiety and Depression Scale (HADS) ^11^. In spite of contradictory findings, the psychological factors are considered to be involved in the pathogenesis of RAS^11–13^. Moreover, 46% of the patients showed a positive familial history of this disorder in a study ^14^, which indicated the genetic role in its pathogenesis.

Approximately, 46% of the patients show a positive familial history ^15^. A variety of causes can play a role in RAS etiology such as local trauma, iron deficiency anemia, folic acid deficiency, vitamin B12 resorption defect ^16^, neutropenia ^17^, and such psychological factors as stress and anger ^13^.

Noticeable evidence has demonstrated that psychological alterations in RAS may be influenced by the serotonergic mechanisms particularly serotonin-transporter gene (*5-HTT*) ^18–20^. The *5-HTT* gene modulates the intensity and duration of serotonergic neurotransmission.Thus, this gene polymorphism can influence the anxiety related behaviors ^21–24^.

The *5-HTT* gene (SLC6A4) is located on chromosome 17q12; it spans 31 *kilobases* (*kb*), and contains 14 exons ^25,26^. Two polymorphic regions have been known on SLC6A4 which are the 44-*base pair* (*bp*) insertion/deletion polymorphism within the promoter region (*5-HTTLPR*), and the 17-*bp* Variable Number of Tandem Repeat (VNTR) polymorphism in intron 2 ^19,25,26^. Two allelic variants, a long (L) 16-repeat allele and a short (S) 14-repeat allele, could be detected on the first polymorphic region (*5-HTTLPR*) ^26^. It has been recently reported that the L and S variants of the promoter polymorphism modulate SLC6A4 differently. The S allele is associated with reduced transcription of SLC6A4 and consequently a reduction in serotonin reuptake ^22^. The investigations have revealed that *5-HTTLPR* could be related to suicidal behaviors ^24,27^, depression ^28^, anger ^29^, and alcoholism ^30^. Several studies have evaluated the correlation between a variety of polymorphisms and aphthous lesions. None of them, however, has assessed *5-HTT* gene polymorphism related to this disease among Iranian population while the 20.7% prevalence of RAS in Iran necessitates it to study various aspects of this issue.

Since psychological and genetic factors influence RAS pathogenesis, the aim of the present study was to evaluate *5-HTTLPR* polymorphism in *5-HTT* gene in patients with RAS in comparison to that in healthy subjects.

## Materials and Methods

### Subjects

This case-control study was conducted on the patients attending the oral medicine department of the International Campus of Dental School, Tehran University of Medical Sciences. One hundred patients with RAS were selected as the case group and one hundred healthy subjects participated as the control group. Both groups were selected from Iranian population. The study process was approved by the Medical Ethics Committee, Office of Vice Chancellor for Research, Tehran University of Medical Sciences.

The inclusion criteria included presence of aphthous lesions which were examined and diagnosed by an oral medicine specialist in the department and recurring episodes of aphthous ulcers at least three times a year according to patient’s history. In this study, 50 male patients and 50 female patients participated.The exclusion criteria included presence of any local oral disease or systemic disorder with oral manifestations including Behcet’s syndrome, Celiac disease, AIDS, pregnancy and the use of immunomodulatory drugs, systemic steroids, or cytotoxic drugs during the last month.

The consent forms were then signed by the study participants or their parents (for the participants up to 18 years old).

A 5 *ml* blood sample was taken from each one of the study participants and was poured in a plastic test tube containing 300 *μl* of ethylene diaminetetraacetic acid (EDTA). The demographic data of the patients, the sampling date, and the sample number were registered on the tubes. The test tubes were then stored in a freezer at −20°*C* until DNA purification time.

### DNA extraction from white blood cells

To extract DNA, 10 *ml* of cold distilled water was poured into each test tube containing frozen blood. The tubes were then placed on a tube rotator for 30 *min* to melt the frozen blood and make the Red Blood Cell (RBC) hemolysis possible.

All blood-contained tubes were then centrifuged at 4500 Relative Centrifugal Force (RCF) for 20 *min* until a red sediment was formed from destroyed RBCs and WBCs at the bottom of the tube. The liquid supernatant was then poured away. Thereafter, 5 *ml* of cold distilled water was poured into the test tube and the process mentioned above was performed for two more times.

Thereafter, Tris buffer 0.606 *ml* (10 *mM*), WB lysis, NaCl 11.688 *g* [400 *mM*], 0.372 *g* EDTA [2 *mM*], SDS (100 *μl*), and proteinase K (200 *μl*) were poured in to each tube and mixed together until the sediment was detached from the bottom of the tube and mixed with the small drop of proteinase k which was probably adhered to tube wall. The tubes were placed in bain-marie (55°*C*), on a shaker for 24 *hr* in order to separate strands of DNA. The test tubes were then centrifuged at 5500 RCF for 30 *min*, and the liquid supernatant was poured into other new tubes. Then, 5 *ml* of cold ethanol was poured into each tube and the white bundle of DNA was gradually formed.

The quality and quantity of the DNA obtained were measured using OD260/OD280 ratio. In this ratio value, 1.8 indicates DNA of high purity, and lower values indicate contamination by RNA ^18^.

### PCR (Polymerase Chain Reaction)

The selective replication from DNA fragments was performed in the presence of a proper buffered solution containing magnesium ions, proper primers, and Taq polymerase enzyme. PCR products were visualized by agarose gel electrophoresis. The gel was stained with ethidium bromide and the analyses were performed ^18^.

**Figure 1.**
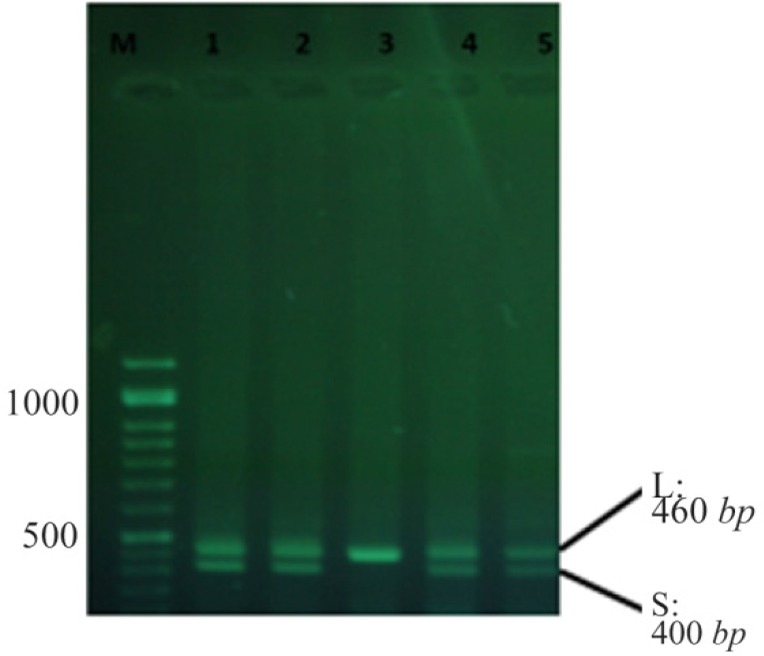
An instance of PCR using primers.

DNA of the patients and healthy control subjects was extracted using a pair of primers capable of distinguishing S/L alleles and replicating *5-HTTLPR*. An instance of PCR by primers is shown in figure 1.

The results of PCR process in case and control groups were then evaluated and all the subjects were divided into three genotype groups: heterozygote with two PCR bands for S and L alleles (LS), homozygote with one PCR band for S alleles (SS), and homozygote with one PCR band for L alleles (LL).

**Figure 2.**
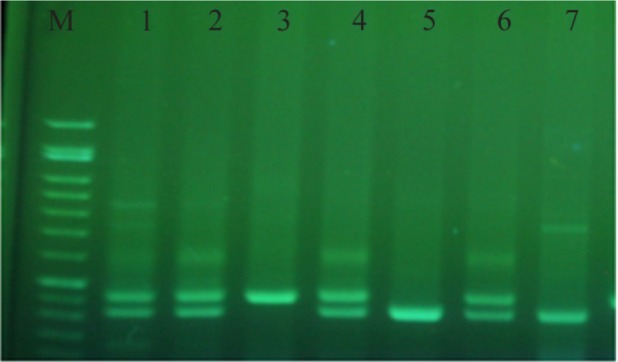
Gel electrophoresis stained with green viewer.

Gel electrophoresis stained with green viewer is shown in figure 2. The DNA size marker is available and the aphthous patient samples are shown in numbers 1 to 7. Numbers 1, 2, 4, and 6 show heterozygote for LS, number 3 shows homozygote for LL, and number 7 shows homozygote for SS.

### Statistical analysis

The qualitative data of the present study were reported by crude and relative frequency. Chi-squared test, or Fisher’s exact test (where necessary) served to compare the distribution of genotype and allele frequencies between two groups. Significance level was set at p<0.05.

Logistic regression analysis served to compare *5-HTT* allele and genotype frequencies between the two groups.

## Results

No statistically significant difference existed in the risk of RAS between the subjects with LL genotype and the subjects with LS genotype (p=0.98) (Table 1).

**Table 1.** The frequency distribution and the relation between the genotype and 5-HTTLPR alleles, the case (n=100) and control (n=100) groups (29)

**Patients (n=100)**		**Control (n=100)**	**Odds Ratio (OR)**	**95%CI**	**p-value**
**Genotype**					
LL	18 (40.91%)	26 (59.09%)	1		
LS	42 (41.18%)	60 (58.82%)	1.01	0.49–2.07	0.98
SS	40 (74.07%)	14 (25.93%)	4.13	1.75–9.71	0.001
**Allele**					
L	78 (41.9%)	112 (58.95%)	1		
S	122 (58.1%)	88 (41.05%)	1.99	1.34–2.96	<0.001

The SS genotype was associated with a 4.13 time higher risk of RAS as compared to the LL genotype, as given (Table 1) (p<0.001). Therefore, having S allele approximately doubled the risk of RAS (p<0.001).

## Discussion

RAS is not recognized as a single disease, but a clinical manifestation of several pathologic conditions such as hematologic and immunologic diseases, stress, and psychological disorders ^7,9,10^.

In previous studies, the levels of depression, anger, and stress in the patients suffering from RAS were determined using Hamilton Anxiety Depression (HAD) scale ^11^, and the psychological factors were demonstrated to be highly associated with RAS pathogenesis ^11–13^. Approximately, 46% of the patients with RAS have a positive family history ^15^. Local trauma, iron deficiency anemia, folic acid deficiency, vitamin B12 resorption defect ^16^, neutropenia ^17^, and psychological factors such as stress and anger ^13^ can play a role in RAS etiology.

The *5-HTT* gene modulates the intensity and duration of serotonergic neurotransmission, thus, this gene polymorphism can influence the anxiety related behaviors ^24,25^. It has been recently reported that the L and S variants of the promoter polymorphism modulate SLC6A4 differently. The S allele is associated with reduced transcription of SLC6A4 and consequently a reduction in serotonin reuptake ^25^. Several studies have evaluated the correlation between a variety of polymorphisms and aphthous lesions; however, none of them has assessed *5-HTT* gene polymorphism related to this disease among Iranian population.

In the present study, 100 patients suffering from RAS and 100 healthy subjects were evaluated. The genotypes of *5-HTTLPR* were divided into three groups by PCR using specific primers: homozygote for SS, homozygote for LL, and heterozygote for LS. L and S allele frequencies showed no statistically significant relation with RAS patients as compared to the healthy subjects. Totally, the results of the present study demonstrated that the frequency of *5-HTTLPR* genotype and L/S alleles could be considered as a risk factor for RAS development and progression.

In 2005, Victoria *et al* evaluated the polymorphism of serotonin transporter gene in 69 patients suffering from RAS and 70 healthy subjects in Brazil. The results of their study showed a significant increase in incidence of SS genotype, S allele, and *5-HTTLPR* polymorphism in the patients with RAS compared to the healthy subjects ^21^.

In 2004, researchers in Michigan University performed a systematic review on the relation between the polymorphism in the promoter region of the serotonin transporter gene (*5-HTTLPR*) and anxiety/ anger related behaviors. They evaluated 23 studies applying meta-analysis. The results revealed a significant relation between the polymorphism of *5-HTTLPR* and neuroticism ^31^.

According to the results of our study, *5-HTTLPR* genotypes and S/L allele frequencies seem to be associated with some of stress-related or auto-immune diseases among Iranian population.

## Conclusion

According to the results of our study, *5-HTTLPR* genotypes and S/L allele frequencies seem to be associated with some of stress-related or auto-immune diseases among Iranian population.
